# Drug-Coated Balloons in Side Branch Treatment in True Coronary Bifurcation Lesions: A Meta-Analysis and Systematic Review

**DOI:** 10.3390/jcm15072489

**Published:** 2026-03-24

**Authors:** Olivia Stainer, Milica Milosavljevic, Kevin Liou

**Affiliations:** 1 Department of Cardiology, Bankstown Hospital, Sydney, NSW 2200, Australia; 2 Faculty of Medicine, University of New South Wales, Sydney, NSW 2052, Australia; 3 Faculty of Medicine, Western Sydney University, Sydney, NSW 2200, Australia

**Keywords:** coronary bifurcation lesions, drug coated balloons, percutaneous coronary intervention

## Abstract

**Background/Objectives**: Coronary bifurcation lesions (CBLs) are common, and the treatments remain nuanced. Side branch (SB) patency is a key determinant of clinical success in CBL intervention. In this paper, data exploring the routine use of drug-coated balloons (DCBs) in the SB is presented as an alternative to standard plain old balloon angioplasty (POBA). **Methods:** A meta-analysis was performed comparing DCBs in the SB to POBA after drug-eluting stent (DES) implantation in the main vessel (MV) of a true CBL. Outcomes including myocardial infarction (MI), target lesion revascularisation (TLR), cardiac death, and late lumen loss (LLL) up to 1 year post-procedure are reported. **Results**: Six studies comprising of 1982 patients were included in the analyses. Patients were predominantly male, and the mean age was >60 years. Four studies included patients with acute coronary syndrome. The primary outcomes were a statistically significant reduction in MI rate (OR 0.38, 95% CI 0.19–0.76, *p* = 0.006), and in the TLR rate (OR 0.45, 95% CI 0.23–0.87, *p* = 0.02) in the DCB group compared to the control. There was no significant difference in cardiac death. Furthermore, there was a significant reduction in SB LLL in the DCB group (Mean difference −0.22mm, 95% CI −0.33–−0.11mm, *p* = 0.0001). **Conclusions:** Following DES implantation in the MV of a true CBL, this analysis demonstrated that DCBs in the SB is superior to POBA in reducing MI, TLR, and LLL of the SB. Large, randomized trials are required to consolidate the role of DCB in the treatment of CBL.

## 1. Introduction

Coronary bifurcation lesions (CBLs) account for up to 20% of all coronary lesions treated with percutaneous coronary intervention (PCI) [1,2,3,4,5,6]. Management of CBL is challenging and associated with worse clinical outcomes compared to non-bifurcation lesions [4,5,6,7]

While treatment approaches vary depending on the anatomy, current guideline supports upfront provisional strategy (PS) in most CBL [8] due to its simplicity, reduced metal burden, and lower complication rates [1,4,6,8]. This approach generally results in better procedural outcomes and fewer long-term adverse events. Unfortunately, angiographic side branch (SB) compromise (e.g., SB occlusion, reduced TIMI flow, dissection, or ostial stenosis) occurs in approximately 10–20% of PS procedures [7,8,9], often leading to poorer patient outcome including higher rates of target lesion failure (TLF), myocardial infarction (MI), and need for reintervention [10].

Bifurcation angle, plaque shift, and ostial SB disease burden are predictors of SB compromise [6,8,11,12,13,14,15]. Selective use of techniques like kissing balloon inflation may help in certain anatomical subsets, although evidence for its routine application particularly with PS is tenuous [16]. Investigations of alternative SB treatment approach is therefore warranted. Emerging therapies such as drug-coated balloons (DCBs) show promise in improving SB outcomes by reducing neointimal hyperplasia without leaving a permanent implant, potentially lowering restenosis rates and preserving vessel anatomy [4,11,12,13,17,18,19,20,21]. The evidence in this space, however, remains scant and incomplete.

This updated meta-analysis aims to inform the current practice regarding the use of routine DCBs for SB treatment in CBL. Previous reviews have included the use of bare metal stent in the main branch and unpublished studies [4,21]. In contrast, this study focuses exclusively on published studies and investigations that reflect contemporary, guideline-directed management strategies where DES was exclusively used in the treatment of main branches.

## 2. Materials and Methods

This was an investigator-initiated study. The study protocol was registered with PROSPERO (CRD42025640738), and the review was conducted in line with PRISMA (Preferred Reporting Items for Systematic reviews and Meta-analyses) guidelines (Supplementary Material).

### 2.1. Search Strategy

A systematic and comprehensive literature search was performed by the two authors (O.S. and M.M.) in July 2025 using Ovid Medline, Embase, Cochrane Register of Controlled Trials (CCTR), and Cochrane Database of Systematic Reviews (CDSR) with no date restriction. The focus was exclusively on human studies published in the English language. The search strategy was (“drug coated balloon” OR “drug-coated balloon” OR “DCB” OR “drug eluting balloon” OR “drug-eluting balloon” OR “DEB”) AND (“coronary” OR “cardiac”) AND “bifurcation lesion” (Supplementary Material, File S1). Furthermore, the reference lists of pre-existing publications on relevant topics were searched for eligible additional articles.

### 2.2. Eligibility Criteria

Studies meeting the following criteria were included in the quantitative analyses: (1) randomized and observational studies directly comparing side-branch DCB use with standard therapy (POBA) after drug-eluting stent (DES) implantation in the main branch of a true coronary bifurcation lesion; (2) studies containing raw data for retrieving directly or permitting indirect derivation of outcome of interests; (3) at least 20 patients were included in the studies.

Studies relevant to the subject of this analysis though not in line with the eligibility criteria, i.e., single-arm studies, were included in the proportional meta-analysis.

Studies were excluded if incomplete data was reported, key outcomes were not reported or if the patients included were not representative of the cohort of interests. Conference abstracts, commentaries, reviews, and duplicated data from the same institutions were similarly excluded from the analyses. Studies which included upfront two stent strategies were also excluded to ensure uniformity of study cohort.

### 2.3. Study Screening and Data Extraction

The studies were retrieved, screened, and selected independently by two of the co-authors (O.S. and M.M.). The data was extracted independently by the same co-authors (O.S. and M.M.) and compiled in separate spreadsheets. Data was compared, and any disagreements in data collected were resolved through consensus and discussion with the senior author (K.L.) [File S1].

### 2.4. Methodological Quality Evaluation

The risk of bias of each study was assessed independently by two authors (O.S. and M.M.) using ROBINS-I for non-randomized studies [Figure S1] and ROB-2 [Figure S2] for randomized studies [22,23]. The outcomes were then discussed amongst the two assessors. The final assessment of each study’s risk of bias was added into Cochrane’s risk of bias assessment tool on Revman to generate a graph for inclusion in the analysis [Figure S3].

### 2.5. Endpoint Definitions

The objective of this study was to compare DCB against standard therapy (POBA) in the management of SB in PS management of CBL. As major adverse cardiovascular events (MACE) were defined differently among included studies, only key and consistently reported components of MACE were included in the analyses.

The primary endpoints included target lesion revascularization (TLR), spontaneous MI, and cardiac death. TLR for the purpose of the present analysis is a summated rate for both the MB and SB. Peri-procedural MI were excluded from analyses due to inconsistent reporting and unclear clinical significance.

The secondary endpoint was LLL at the SB, defined as the difference between the post-procedural minimal luminal diameter (MLD) and the MLD measured at angiographic follow-up. Pooled event rates for each individual outcome are also estimated.

Specific endpoint definitions for each study are presented in Table 1 and Table S3.

### 2.6. Statistical Analysis

Study-level analyses were performed with Review Manager (RevMan, The Cochrane Collaboration, Ver 5.4). The Wald method was employed with a random effect model, chosen a priori due to the anticipated heterogeneity among the included studies. Data was analyzed using an inverse variance model to increase precision of the overall effect. The measure of relative treatment effect was expressed in odds ratio (OR) accompanied by 95% confidence intervals (CI). Where there were zero events in one of the comparison arms, continuity correction was employed to derive the OR.

Proportional meta-analysis was performed with MetaAnalysisOnline (https://metaanalysisonline.com, Accessed on 30 July 2025) across both single- and double-arm studies to ascertain the overall rate of TLR, cardiac mortality, and MI.

A trend was considered statistically significant if the *p* value was less than 0.05 and the CI did not cross neutrality. Heterogeneity among studies was examined with the Restricted Maximum-Likelihood (REML) and I^2^ statistic, with *p* < 0.10 indicating the presence of significant heterogeneity, and I^2^ values of 25, 50, and 75% corresponding to low, moderate, and high degrees of heterogeneity. Publication bias was assessed based on study distribution on the funnel plot [Figure S4A–E].

## 3. Results

**Figure 1:**
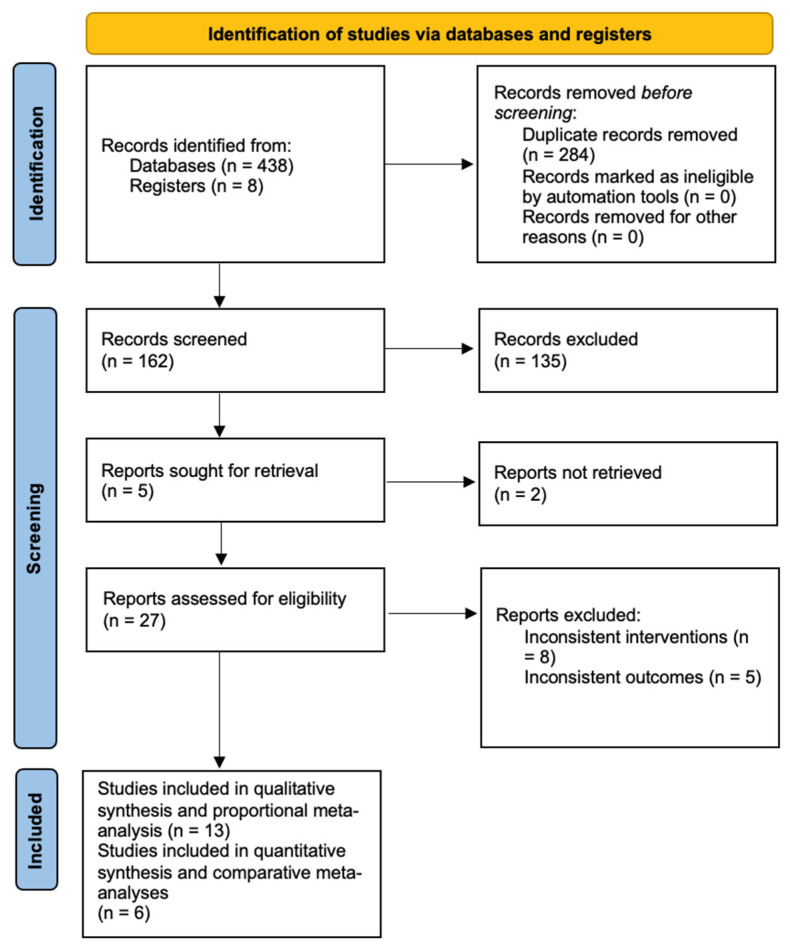
PRISMA flow diagram of studies included and excluded from systematic review and single arm proportional meta-analysis.

The study selection process was outlined in the PRISMA flow diagram (Figure 1).

Among the 13 remaining studies, 6 peer-reviewed publications were included for comparative quantitative analyses. Overall, 1982 patients were included. Among them, 885 (44.7%) patients underwent DCB treatment. The follow-up duration ranged from 6–24 months.

The study, lesion, and procedural information are presented in Table 1, Table 2 and Table 3. The patient information and summary of outcomes are presented in Supplementary Tables S1 and S2.

### 3.1. Primary Endpoints

**Figure 2:**
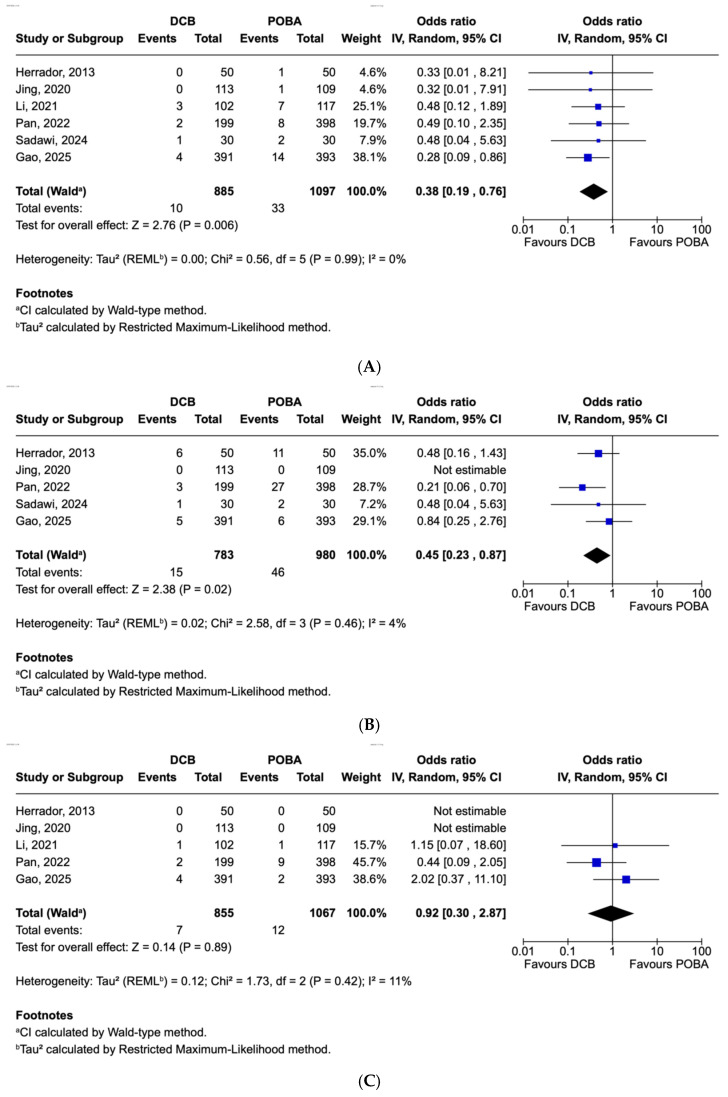

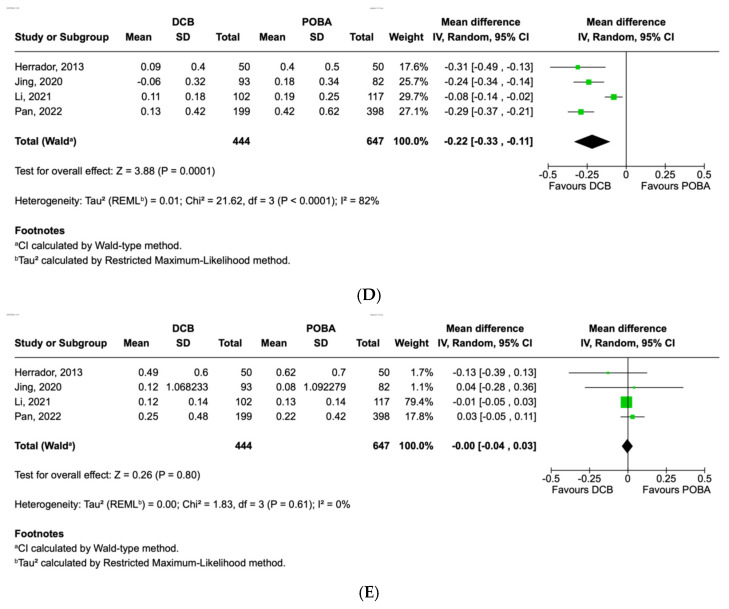
(**A**): Myocardial infarction. Rates of myocardial infarction in patients with coronary bifurcation lesions treated with drug-eluting stent in the main vessel, and either plain old balloon angioplasty (POBA) (control) versus drug-coated balloon (DCB) (experimental) in the side branch [11,13,14,24,25,26]. (**B**): Target lesion revascularization. Rates of target lesion revascularization in patients with coronary bifurcation lesions treated with drug-eluting stent in the main vessel, and either plain old balloon angioplasty (POBA) (control) versus drug-coated balloon (DCB) (experimental) in the side branch [11,13,14,25,26]. (**C**): Cardiac mortality. Rates of cardiac mortality in patients with coronary bifurcation lesions treated with drug-eluting stent in the main vessel, and either plain old balloon angioplasty (POBA) (control) versus drug-coated balloon (DCB) (experimental) in the side branch [11,13,14,24,25]. (**D**): Late lumen loss side branch at follow up. Late lumen loss of the side branch of coronary bifurcation lesions treated with a drug-eluting stent in the main vessel, and side branch treated with either plain old balloon angioplasty (POBA) (control) versus drug-coated balloon (DCB) (experimental) [11,13,14,24]. (**E**): Late lumen loss main branch at follow up. Late lumen loss of the main branch of coronary bifurcation lesions treated with a drug-eluting stent in the main vessel, and side branch treated with either plain old balloon angioplasty (POBA) (control) versus drug-coated balloon (DCB) (experimental) [11,13,14,24].

There was a statistically significant trend in favor of DCB (OR 0.38, 95% CI 0.19–0.76 *p* = 0.006) with respect to the rate of MI at follow-up (Figure 2A). There was minimal heterogeneity among the included studies (I^2^ = 0% *p* = 0.99).

There was a statistically significant reduction in TLR rate in the DCB group (OR 0.45, 95% CI 0.23–0.87, *p* = 0.02) compared with standard treatment (Figure 2B).

In studies where outcomes were reported, there was no significant difference in cardiac mortality between the two treatment arms (Figure 2C).

### 3.2. Secondary Endpoints

There was a significant reduction in LLL in the DCB group compared to the POBA group in the SB (Figure 2D). The mean MLD difference in LLL was −0.22mm (*p* = 0.0001). The I^2^ of the studies was high at 82 indicating heterogeneity between the results, although the trend in favor of DCB was consistent. There was no significant difference in LLL of the MB at follow up (Figure 2E).

### 3.3. Proportional Meta-Analysis

The overall rate of MI, TLR, and cardiac mortality at 12 months across all studies were 0.01 (0.00–0.02) (8 studies, 1384 participants), 0.02 (0.01–0.04) (7 studies, 1282 participants), and 0.00 (0.00–0.01) (8 studies, 1384 participants), respectively (Figure S5A–C).

## 4. Discussion

The present study is distinguished by its focus on true bifurcation lesions under a hybrid DES-DCB framework and included both randomized and real-world data. The main findings of this study are that, in a contemporary cohort of patients with true CBL undergoing PCI, the use of DCB in SB over conventional POBA resulted in a statistically and clinically relevant reduction in recurrent spontaneous MI, TLR, and LLL of the SB. The absolute event rates for MI, TLR, and cardiac mortality are low by contemporary standards.

### 4.1. Shortfalls of Current CBL Treatments

Despite progress made in recent years, significant limitations remain in the treatment of true CBL. Even when optimized, bifurcation PCI continues to be associated with high MACE rates in both left main and non-left main CBL [4,6,7,11,13,14,27,28].

In EBC MAIN (*n* = 467) [29], the provisional stenting arm demonstrated a composite event rate of 14.7% at one year, including 7.1% TLR and a 5.1% all-cause mortality rate. The DKCRUSH V (*n* = 482) trial [30] showed that DK crush for complex left main bifurcations resulted in a target lesion failure rate of 8.3% and a stent thrombosis rate of 2.7% at one year.

In non-left main bifurcations, the provisional arm of the EBC TWO trial showed a 12-month MACE rate of 7.7% and a TVR rate of 2.9% [31]. Similarly, in the COBIS II registry, the MACE and TLR rates were 6.8% and 5.8%, respectively, in those treated with provisional strategy followed by routine final kissing inflation (FKI). The rates were even higher if FKI was not attempted at 8.6% and 6.6%, respectively [32].

In the present analysis, the incorporation of DCB in the treatment of true CBL appears to result in lower recurrent event rates. The proportional meta-analysis demonstrated a summated MI and TLR rate of 1 and 2% at 12 months, respectively. Notwithstanding the differences in study design, these event rates are low by contemporary standard. This highlighted the fact that DCB in the SB a feasible and clinically relevant alternative to conventional therapy in the treatment of true CBL.

### 4.2. Reduction in LLL Compared to Conventional Therapy

The present analyses demonstrated a significant reduction in LLL in the SB when comparison was made to POBA. DCBs work by delivering and permeating antiproliferative drug, most commonly paclitaxel, into the vessel wall. The primary mechanism by which paclitaxel attenuates LLL in bifurcation lesions, especially in the SB, is by preventing neointimal hyperplasia and excessive smooth muscle cell proliferation [33]. These processes are central to the pathogenesis of neointimal hyperplasia and restenosis following coronary interventions. There is also evidence correlating the use of DCB and positive endothelial remodeling, which in some studies was found to increase lumen diameter over time [17,34].

While beyond the scope of the present analysis, DCB may also offer theoretical advantages over two stent treatment to complex CBL. DCB offers a more homogeneous and efficient delivery of antiproliferative drugs to the coronary artery wall compared to DES, owing to distinct differences in their drug delivery mechanisms and pharmacokinetics. Unlike DES, which release drugs gradually from a polymer-coated metallic scaffold over weeks or months, DCBs deliver the drug through a single, high-concentration burst during balloon inflation. This allows for rapid drug transfer and uniform uptake into the vessel wall [17,35]. The absence of a metallic scaffold in DCBs eliminates structural barriers to drug diffusion. In addition, DCBs enable drug delivery across the entire treated segment, including areas that may be difficult to access with stent struts, such as curved vessel segments, thus ensuring more even and complete antiproliferative coverage. This is particularly advantageous in bifurcation lesions, where the complex geometry and flow patterns can limit optimal stent expansion and drug distribution.

There was no significant change in the LLL of the main branch, which is expected given DES were used in both the experimental and control groups.

### 4.3. Reduction in TLR and Spontaneous MI

The reduction in LLL of the SB is likely the mechanism by which DCB use results in a reduction in TLR and spontaneous MI. The ability of DCB to reduce restenosis and promote late positive vessel remodeling likely contribute to a lower risk of TLR compared to traditional balloon angioplasty. The consequent attenuated disruption to the endothelium, plaque rupture, and thrombus formation leads to reduction in spontaneous MI. The interpretation of this trend should be with caution due to the small absolute number of events and the likely fragility of pooled estimates. However, the recently published ANDROMEDA study [36] signaled potential benefit in spontaneous MI reduction following DCB treatment to small vessels compared with DES. This bears relevance particularly when an upfront two stent strategy is contemplated.

### 4.4. Future Direction

#### 4.4.1. Stentless Approach to Treatment of CBL

The drug-coated balloon (DCB)-only approach for the treatment of true CBL has garnered increasing interest due to its potential to avoid vessel caging and associated long-term complications. Several studies have explored the feasibility and clinical efficacy of this strategy in selected bifurcation settings. The PEPCAD-BIF trial [34] was among the first prospective studies to evaluate DCB-only angioplasty in coronary bifurcations, including true bifurcation lesions. In this trial, which enrolled 64 patients, the in-segment LLL at 9 months was 0.22 ± 0.50 mm in the DCB group versus 0.11 ± 0.45 mm in the paclitaxel-eluting stent group, demonstrating non-inferiority. TLR occurred in 6.3% of the DCB group, indicating acceptable safety and efficacy when optimal lesion preparation was performed.

In a prospective multicenter study performed by Hu et al., DCB only approach to the treatment of 66 bifurcations lesions led to a TLR and MI rate of 0% and 1.5%, respectively [37].

These findings underscore the merit of a DCB-only strategy in reducing foreign body implantation, preserving vessel anatomy, and potentially shortening dual antiplatelet therapy duration, particularly in small vessel bifurcations and high bleeding risk patients. However, despite these encouraging outcomes, the current body of evidence remains limited by small sample sizes, non-randomized designs, and heterogeneity in lesion complexity and procedural technique. Critically, there is a lack of large-scale randomized controlled trials focused specifically on true bifurcation lesions treated with a DCB-only strategy. This constitutes a significant gap in knowledge, and ongoing trials are expected to provide more definitive evidence to guide future clinical practice.

#### 4.4.2. Optimal Treatment Approach with DCB to the SB

The optimal DCB deployment algorithm has not been clearly defined in the context of true CBL hybrid treatment with PS. The authors advocate SB predilatation prior to MB stenting. During MB stent optimization, SB ostial dilatation should be routinely performed to prevent stripping of drug coating as a DCB is advanced through undilated side struts. The DCB is then deployed into the SB, before FKI is performed with a non-compliant (NC) balloon in the MB and the DCB, which is partially withdrawn into the MB. Alternatively, FKI may also be performed with an NC balloon in the SB [6]. Proximal optimization should then be performed. Intracoronary imaging should be deployed where feasible, while specialty balloons may be considered. This approach however requires validation in future trials.

### 4.5. Limitations

Several limitations merit consideration. First, this was a study level meta-analysis. The reliance on aggregate rather than individual patient data precludes adjustments for patient-level confounders such as age, comorbidities, clinical presentations, as well as procedural and lesion characteristics.

The overall number of studies included was small, reflecting the lack of evidence base in this space. This may affect the strength of causal inferences. In contrast to previous meta-analysis [4], however, the authors elected to included only published studies and those in whom DES, and not BMS, were used in the main vessels to maintain the integrity of the data and its relevance to modern practice. It is noted that this would affect the publication bias assessment by funnel plots.

Three of the included studies were observational in nature. The impact of selection bias and operator discretions could not be eliminated, especially as the ROBINS-I risk of assessment disclosed moderate risks of biases across these three studies. However, it is worth noting that, within each study, the baseline clinical and angiographic characteristics were relatively balanced. The event rates in the control groups were similar to previously published studies, suggesting that the present cohort was likely representative and contemporary. It is acknowledged, nevertheless, that the heterogeneity in study methodology should be taken into consideration when the results are interpreted. Indeed, the authors also identified moderate degree of biases across the RCT based on the ROB-2 Risk Assessment Tool.

The use of intracoronary imaging and cutting or scoring balloons was not routine among the included studies. It is unclear, however, if this would have impacted on the analysis significantly. The role of intracoronary imaging in DCB intervention is not well established. While it may facilitate lesion characterization and vessel sizing, imaging endpoints following DCB intervention has not been clearly defined. In the present analysis, studies that used imaging have not demonstrated clear advantage over those which did not.

Lesion modification with cutting and scoring balloons may improve drug diffusion, although no outcome studies have been performed in this regard particularly in the context of SB intervention, where the vessels are often small and there may be a higher risk of vessel disruption necessitating bail out stenting.

There is no class effect for DCB. They vary by design, excipients, coating as well as drug type and concentrations. The types of DCB used among the included studies were not uniform or consistently reported. Caution is therefore required to generalize the results of the present analyses to DCB across the spectrum.

Further, in Pan et al., around half of the patients in the control group received a DES to the SB, which may have skewed the result in favor of the treatment group.

These differences should ideally be explored with various sensitivity analyses. However, given the small overall sample size, it is unclear if the results would have been meaningful with further segmentation of available data.

The authors also wish to draw attention to the differences in follow-up timing. In interpreting the data, recommendation is made to focus on the directionality and not necessarily the effect size estimate.

The I^2^ value is high for LLL for the SB. This is not uncommon in meta-analyses of continuous data particularly when sample size is small. In this context, it tends to indicate the proportion of total variance attributed to heterogeneity and not the actual magnitude of variance. Again, the authors wish to highlight the directionality and not the effect size of the trend, where all included studies unequivocally demonstrated benefits in favor of DCB use. Had the analyses been rendered binary in nature, it is expected that the I^2^ value would greatly diminish.

## 5. Conclusions

Following DES implantation in the MV in a CBL, the present analysis demonstrated that DCB treatment in the SB may lead to superior outcome compared with standard therapy in terms of recurrent MI, TLR, and LLL of the SB. The result is hypothesis generating and large, randomized trials are required to determine if this hybrid approach should be routinely incorporated into the treatment of CBL.

## Figures and Tables

**Table 1:** Studies included in the meta-analysis.

Study, Design, and Recruitment	Intervention	Inclusion Criteria	MV Stent	SB DCB	Primary Endpoints	Secondary Endpoints	Follow Up and Loss to Follow Up
**Herrador, 2013 [14]** Prospective comparative study Single center January 2009–March 2011	(A) DES in MV + POBA in SB (*n* = 50) (B) DES in MV + DEB in SB (*n* = 50)	(1) Lumen of both vessels ≥2.5 mm; (2) SB stenosis length <10 mm; (3) Any medina classification	Taxus Liberté^®^	SeQuent^®^ Please	Angiographic results—binary restenosis and LLL	(1) MACE (2) US measurements differences in the MV and SB immediately after PCI and at f/up	Phone call @ 12 mo: 100% phone review f/up Angiographic imaging @ 12 mo: refusal @ 12 mo: (A) 7 (B) 10
**Jing, 2020 [11]** Prospective, multicenter, randomized study 10 Chinese centers December 2014–November 2015	(A) DES in MB, DEB in SB (*n* = 113) (B) DES in MB, POBA in SB (*n* = 109)	M + F, age 18–80 years with angina pectoris, an old MI or evidence of asymptomatic myocardial ischemia. MB de novo CBL. Diameter stenosis of the SB ≥ 70% by visual estimate and residual stenosis 50% after pre-dilation by regular balloon dilation. The target lesion had to have a reference vessel diameter ≥1.25 and 5.00 mm and a lesion length 40 mm.	DES-unspecified	Bingo^®^	Angiographic target lesion stenosis at 9 mo	(1) LLL at 9 months; TLR, TVR, TLF, MACCEs, all-cause death, cardiac death, non-fatal MI, and thrombosis in target lesions	Outpatient or telephone follow-up at 30, 180, and 270 days after surgery. Phone loss to follow up: (A) 0, (B) 1 Angiographic follow-up at 270 days after surgery. A total of 47 people refused angiography follow-up 270 days after surgery.
**Li, 2021 [24]** Retrospective cohort study Single center September 2016–March 2019	(A) DES in MV + POBA of SB (*n* = 117) (B) DES in MV + DEB in SB (*n* = 102)	(1) de novo bifurcation disease, lumen stenosis ≥ 50%; (2) age ≥ 18 years old; (3) true CBL (Medina classification (1,1,1), (1,0,1), (0,1,1)); (4) residual stenosis < 50% after pre-dilation	Everolimus-coated DES (Promus Premier)	SeQuent^®^ Please	MACE	MLD, LLL, and restenosis for MV and SB	12-month follow-up for angiography and cardiovascular events after PCI. No loss to follow up.
**Pan, 2022 [13]** Prospective non-randomized control trial 3 centers June 2015–May 2019	(A) DES in MB + DCB in SB (*n* = 199) (B) DES-only strategy (provisional stenting or two-stent strategies) (*n* = 398). 173 patients (43.47%) treated with 1-stent strategy.	Patients with angina with de novo coronary lesions (diameter stenosis > 50%) at the LM bifurcation (Medina (1,0,1), (0,1,1) or (1,1,1)), with an SB diameter ≥ 2.0 mm	Not specified	SeQuent^®^ Please	2-year composite rate of TLF: cardiac death, TVMI, or CD-TLR	All-cause mortality, cardiac death, TVMI, CD-TLR, and stent thrombosis (ST)	For 2 years, 3 monthly office visits or telephone calls. Coronary angiography @ 12 months post-PCI, unless earlier clinical indication. Angiographic follow-up was completed in 66% (394/597) of patients.
**Gao, 2025 [25]** Multicentere randomized controlled trial 22 centers 8 September 2020–2 June 2023	(A) DCB in SB + DES in MV (*n* = 391) (B) NCB in SB + DES in MV (*n* = 393)	(1) Age > 18 years, (2) silent ischemia, stable or unstable angina, or AMI older than 1 week from the onset of chest pain to admission. Target lesion criteria: (1) vessel diameter by visual estimate (both MV and SB) of ≥2.5 mm, (2) baseline diameter stenosis of ≥50%, (3) SB lesion length of <10 mm, (4) successful recanalization of a chronic total occlusion in either the MV or SB before enrolment, and (5) ostial SB diameter stenosis of ≥70% after stenting the MV	Not specified	Not specified	MACE	All-cause death or cardiac death, MACE without periprocedural MI, periprocedural and spontaneous MI, TVMI, CD-TLR, or TVR, angiographic and clinical/procedural success, and crossover from 1 stent to 2 stents	1 year follow up. No loss to follow up at 1 year.
**Sadawi, 2024 [26]** RCT single centere July 2022–Jan 2024	(A) DES in MB, POBA in SB (*n* = 30) (B) DES in MB, DCB in SB (*n* = 30)	(1) Age > 18 years, (2) indicated for elective coronary angiography according to ESC 2018 guidelines with (3) bifurcation lesion affected side branch (SB), (4) planned for provisional stenting technique from the start or shifted to 2-stent strategy as a bailout to the side branch and (5) bifurcation lesion with Medina classification (1,1,1), (1,0,1), and (0,1,1).	Ultimaster, Resolute, onynx, Xinece alpine, Promus	Not specified	MACE	Lesion success and procedure success. Occurrence of any complication to the side branch aborting the procedure and shifting to standard 2-stent techniques	6mo at outpatient clinic; clinical follow up for MACE (a) 2 (6.67%) lost to follow up; (b) 0%

DES: Drug-eluting stent; MV: Main vessel; POBA: Plain old balloon angioplasty; SB: Side branch; DEB: Drug-eluting balloon; LLL: Late lumen loss; MACE: Major adverse cardiac event; US: Ultrasound, PCI: Percutaneous coronary intervention; MI: Myocardial infarction; CBL: Coronary bifurcation lesion; TLR: Target lesion revascularization; TVR: Target vessel revascularization; MLD: Minimum lumen diameter; LM: Left main; TLF: Target lesion failure; TVMI: Target vessel myocardial infarction; CD-TLR: Clinically driver target lesion revascularization; AMI: Acute myocardial infarction; RCT: Randomized control trial; M: Male; F: Female; mo: months; MACCE: Major adverse cardiac and coronary events.

**Table 2:** Lesion characteristics.

Study Characteristics	Medina Classification of Lesions	Number of Diseased Vessels	Lesion Locations	SB Reference Vessel Size (mm)	MV Stent Length (mm)	DCB Diameter and Length (mm)
**Herrador, 2013 [14]**	C: x,x,1 25 (50%) E: x,x,1 32 (64%)	C: Multivessel disease: 62% E: Multivessel disease: 62%	C: LAD/Diagonal 58%, CX/MO 24%, PDA/PL 6%, LMCA 12% E: LAD/Diagonal 50%, CX/MO 26%, PDA/PL 14%, LMCA 8%, Other 2%	C: 2.6 ± 0.2 E: 2.6 ± 0.3	C: 23.8 ± 7.9 E: 22.2 ± 6.4	Diameter: 2.6 ± 0.2 Length: 13.9 ± 3
**Jing, 2020 [11]**	C: M111 94.4%, M101 2.8%, M001 2.8% E: M111 89.4%; M101 4.4%, M011 6.2%	Not specified	C: MB LAD 78%, MB LCX: 17%, MB RCA 4%, SB D:85%, SB OM 12% E: MB LAD 75.2%, MB LCX 13.3%, MB RCA 8%, SB D 73.7%, SB OM 10.5%	C: 2.12 ± 0.28 E: 2.14 ± 0.32	C: 28.0 (23.0–33.0) E: 24.0 (21.0–30.0)	Diameter: 2.50 (2.00–2.50) Length: 15.0 (15.0–20.0)
**Li, 2021 [24]**	C: 111 18%; 101 73.5%; 011 8.6% E: 111 26.5%; 101 56.9%; 011 16.7%	Not specified	LM/LAD/LCx C: 39.3%; E: 58.8%, LAD/Diagonal C: 52.1%; E: 36.3%, LCx/OM C: 8.6%; E: 4.9%	C: 2.47 ± 0.38 E: 2.52 ± 0.35	Not specified	Not specified
**Pan, 2022 [13]**	C: 011 11.31%; 111 79.40%; 101 9.30% E: 011 8.04%; 111 83.92%; 101 8.04%	C: Multivessel disease 87.44% E: Multivessel disease: 87.94%	100% lesions: left main bifurcation lesions	C: 2.98 ± 0.32 E: 3.00 ± 0.49	C: 36.49 ± 13.19 E: 38.69 ± 15.05	Diameter: 2.88 ± 0.39 Length: 18.84 ± 5.22
**Gao, 2025 [25]**	C: 101 6.6%; 011 16%; 111 77.4% E: 101 6.4%; 011 18.7%; 111 74.9%	C: 1 vessel: 32.8%; 2 vessel: 40.5%; 3 vessel: 26.7% E: 1 vessel: 36.6%; 2 vessel: 39.1%; 3 vessel: 24.3%	C: Distal LM 14.2%; LAD 69.5%; LCx 11.7%; RCA 4.6% E: Distal LM 16.1%; LAD 66.5%; LCx 11%; RCA 6.4%	Not specified	C: 42.13 ± 21.38 E: 41.97 ± 19.99	Not specified
**Sadawi, 2024 [26]**	C: 111 53.3%; 101 23.3%; 011 23.3% E: 111 70.0%; 101 10.0%; 011 20.0%	C: 2 vessels: 66.7%, 3 vessels: 33.3% E: 2 vessel: 63.3%, 3 vessels: 36.7%	Not specified	Not specified	C: 30.6 ± 8.77 E: 27.8 ± 8.9	Not specified

C: Control; E: Experimental; SB: Side branch; DCB: Drug-coated balloon; LAD: Left anterior descending; CX: Circumflex; MO; Marginal obtuse; PDA: Posterior descending artery; PL: Posterior-lateral branch of the right coronary artery; LMCA: Left main coronary artery, RCA: Right Coronary Artery; D: Diagonal branch; OM: Obtuse marginal branch; LM: Left Main.

**Table 3:** Procedural Characteristics.

Study Characteristics	KISS Inflation	POT Performed	DAPT Regime	Use of IVUS and/or OCT
**Herrador, 2013 [14]**	Yes—post DCB use	No	DAPT 12 months post-procedure (aspirin 100mg OD + clopidogrel 75 mg OD)	IVUS used
**Jing, 2020 [11]**	Yes—post DCB use	No	DAPT; aspirin and clopidogrel/ticagrelor for at least 12 months post-procedure	Nil IVUS
**Li, 2021 [24]**	No	No	Not specified—100% of patients on DAPT prior to procedure	Nil IVUS
**Pan, 2022 [13]**	Yes	Yes	Not specified	IVUS used
**Gao, 2025 [25]**	Yes—post DCB use	Yes	DAPT-P2Y12 receptor inhibitor and aspirin were prescribed for 12 months—used in 355 (90.8%) patients in the DCB group and 356 (90.6%) patients in the NCB group	IVUS used, OCT used
**Sadawi, 2024 [26]**	No	Yes	DAPT regime and duration prescribed as per ESC 2018 guidelines	Nil IVUS

POT: Proximal Optimization Technique; DAPT: Dual Antiplatelet Therapy; IVUS:, Intravascular Ultrasound; OCT: Optimal Coherence Tomography; OD: Once Daily; DCB: Drug-Coated Balloon.

## Data Availability

The original contributions presented in this study are included in the article/Supplementary Materials. Further inquiries can be directed to the corresponding authors.

## Supplementary Materials

The following supporting information can be downloaded at: https://www.mdpi.com/article/10.3390/jcm15072489/s1, PRISMA 2020 Checklist; PRISMA 2020 Abstract Checklist [38]; File S1: Search strategy, Figure S1: ROBINS-I Risk of Bias Assessment, Figure S2: ROB-2 Risk of Bias Assessment, Figure S3: Assessment of Bias tool; Figure S4: (A): Cardiac mortality at follow up funnel plot, (B): late lumen loss MB at follow up, (C): late lumen loss SB at follow up, (D) myocardial infarction at follow up, (E): target lesion revascularization at follow up; Figure S5: Proportional Meta-analysis; Table S1: Patient characteristics, Table S2: Summary of study outcomes, Table S3: Single-arm characteristics.

## Abbreviations

The following abbreviations are used in this manuscript:

CBL	Coronary bifurcation lesion
DCB	Drug-coated balloon
DES	Drug-eluting stent
LLL	Late lumen loss
MI	Myocardial infarction
MV	Main vessel
POBA	Plain old balloon angioplasty
SB	Side branch
TLR	Target lesion revascularization
