# Supplementary material for: Drug-Coated Balloons in Side Branch Treatment in True Coronary Bifurcation Lesions: A Meta-Analysis and Systematic Review
Source: J Clin Med. 2026 Mar 24;15(7):2489. doi: 10.3390/jcm15072489 (PMC13073584; doi:10.3390/jcm15072489)
Supplement: Supplementary file 1 [file jcm-15-02489-s001.zip › jcm-4188856-supplementary.pdf]

Supplementary materials

Drug coated balloon in side branch treatment of true coronary bifurcation lesions: a focused meta-analysis and systematic review

Table of Contents

PRISMA 2020 Checklist 3

File S1: Search strategy 9

Search Hedge 9

Databases 9

Figure S1: ROBINS-I Risk of Bias Assessment Table 10

Figure S2: ROB-2 Assessment of Bias Table 11

Figure S3: Assessment of Bias 12

Table S1: Patient Characteristics 13

Table S2: Summary of Study outcomes 14

Figure S4: Funnel Plot 15

Figure S5. Proportional Meta-analysis 18

Table S3: Single Arm Study Characteristics 19

PRISMA 2020 Checklist

Section and Topic	Item #	Checklist item	Location where item is reported
**TITLE**			
Title	1	Identify the report as a systematic review.	Stated in the title and outlined in paragraph 4 of the Introduction section.
**ABSTRACT**			
Abstract	2	See the PRISMA 2020 for Abstracts checklist.	
**INTRODUCTION**			
Rationale	3	Describe the rationale for the review in the context of existing knowledge.	Outlined in the Introduction section
Objectives	4	Provide an explicit statement of the objective(s) or question(s) the review addresses.	Outline in paragraph 4 in the Introduction section
**METHODS**			
Eligibility criteria	5	Specify the inclusion and exclusion criteria for the review and how studies were grouped for the syntheses.	Outlined in Eligibility Criteria in the Methodology section
Information sources	6	Specify all databases, registers, websites, organisations, reference lists and other sources searched or consulted to identify studies. Specify the date when each source was last searched or consulted.	Outlined in Search Strategy in the Methodology section
Search strategy	7	Present the full search strategies for all databases, registers and websites, including any filters and limits used.	Outlined in Page 3 of Supplementary Material and referred to in Search Strategy, paragraph 1, in the Methodology section
Selection process	8	Specify the methods used to decide whether a study met the inclusion criteria of the review, including how many reviewers screened each record and each report retrieved, whether they worked independently, and if applicable, details of automation tools used in the process.	Outlined in Methodological Quality Evaluation and Data Extraction in the Methodology section. No automation tools were used in the process.
Data collection process	9	Specify the methods used to collect data from reports, including how many reviewers collected data from each report, whether	Outlined in Methodological Quality Evaluation and Data Extraction in the Methodology section. The authors did not attempt to contact the original

		they worked independently, any processes for obtaining or confirming data from study investigators, and if applicable, details of automation tools used in the process.	study investigators although it is recognized that this could be valuable where published data was incomplete. No automation tools were used in the process.
Data items	10a	List and define all outcomes for which data were sought. Specify whether all results that were compatible with each outcome domain in each study were sought (e.g. for all measures, time points, analyses), and if not, the methods used to decide which results to collect.	Outlined in Endpoint Definition in the Methodology Section. It is recognized that the time points at which the outcomes were assessed differed between studies. No attempts therefore were made to summarise the effect sizes in terms of rates or incidences of a particular outcome, but rather odds ratios.
	10b	List and define all other variables for which data were sought (e.g. participant and intervention characteristics, funding sources). Describe any assumptions made about any missing or unclear information.	Outlined in Tables 1, 2 and 3. Where the variables were not clearly stated, they were not included in the tabulation process and no assumptions were made in this regard.
**Study risk of bias assessment**	11	Specify the methods used to assess risk of bias in the included studies, including details of the tool(s) used, how many reviewers assessed each study and whether they worked independently, and if applicable, details of automation tools used in the process.	Outlined in Methodological Quality Evaluation and Data Extraction in the Methodology Section. No automation tools were used.
**Effect measures**	12	Specify for each outcome the effect measure(s) (e.g. risk ratio, mean difference) used in the synthesis or presentation of results.	Outlined in Statistical Analysis in the Methodology Section.
**Synthesis methods**	13a	Describe the processes used to decide which studies were eligible for each synthesis (e.g. tabulating the study intervention characteristics and comparing against the planned groups for each synthesis (item #5)).	Outcomes reported by each eligible study were tabulated (Table 1). Studies containing various outcomes of interests were included for each individual synthesis.
	13b	Describe any methods required to prepare the data for presentation or synthesis, such as handling of missing summary statistics, or data conversions.	Data syntheses and presentations were automatically performed with Review Manager (Ver 5.4) as outlined in Statistical Analysis in the Methodology section. Where outcomes were reported as percentages, conversion to whole numbers was achieved by multiplying the percentages by the number of patients in each arm
	13c	Describe any methods used to tabulate or visually display results of individual studies and syntheses.	Data syntheses and presentations were automatically performed with Review Manager (Ver 5.4) as outlined in Statistical Analysis in the Methodology section

	13d	Describe any methods used to synthesize results and provide a rationale for the choice(s). If meta-analysis was performed, describe the model(s), method(s) to identify the presence and extent of statistical heterogeneity, and software package(s) used.	Outlined in Statistical Analysis in the Methodology section as well as the abstract.
	13e	Describe any methods used to explore possible causes of heterogeneity among study results (e.g. subgroup analysis, meta-regression).	Outlined in Statistical Analysis in the Methodology section.
	13f	Describe any sensitivity analyses conducted to assess robustness of the synthesized results.	Outlined in Statistical Analysis in the Methodology section.
**Reporting bias assessment**	14	Describe any methods used to assess risk of bias due to missing results in a synthesis (arising from reporting biases).	A funnel plot was used to assess reporting biases and presented in Supplementary Figure S2. The impact of potential publication bias is discussed in Assessment of Certainty in Body of Evidence in the Supplementary Material
**Certainty assessment**	15	Describe any methods used to assess certainty (or confidence) in the body of evidence for an outcome.	Outlined in Statistical Analysis in the Methodology section.
**Section and Topic**	**Item #**	**Checklist item**	**Location where item is reported**
**RESULTS**			
Study selection	16a	Describe the results of the search and selection process, from the number of records identified in the search to the number of studies included in the review, ideally using a flow diagram.	Please refer to Figure 1.
	16b	Cite studies that might appear to meet the inclusion criteria, but which were excluded, and explain why they were excluded.	Please refer to Figure 1.
Study characteristics	17	Cite each included study and present its characteristics.	Please refer to Table 1.
Risk of bias in studies	18	Present assessments of risk of bias for each included study.	Outlined in Supplementary Material.
Results of individual studies	19	For all outcomes, present, for each study: (a) summary statistics for each group (where appropriate) and (b) an effect estimate and its precision (e.g. confidence/credible interval), ideally using structured tables or plots.	Please refer to Figures 2 and 3.

Results of syntheses	20a	For each synthesis, briefly summarise the characteristics and risk of bias among contributing studies.	Please refer to Figures 2 and 3. Risk of Bias has been outlined in the Supplementary Material.
	20b	Present results of all statistical syntheses conducted. If meta-analysis was done, present for each the summary estimate and its precision (e.g. confidence/credible interval) and measures of statistical heterogeneity. If comparing groups, describe the direction of the effect.	Please refer to Figures 2 and 3.
	20c	Present results of all investigations of possible causes of heterogeneity among study results.	The only outcome where significant heterogeneity in results was observed was cardiovascular death. However, given the small number of studies included in this synthesis a subgroup analysis could not be performed to explore this heterogeneity further.<br><br>Significant intergroup heterogeneity was identified in TLR with subgroup analyses based on IVUS use as well as whether final balloon kissing inflation was mandated. Please refer to Figures 2D, 2E and 3C.
	20d	Present results of all sensitivity analyses conducted to assess the robustness of the synthesized results.	Two sensitivity analyses were performed. The first was to examine the impact exclusion of circumflex restenosis on the treatment effects as outlined in paragraph 2 of Primary End Point in the Result section. The second involved the exclusion of one study that included exclusively high-risk patients, which was outlined in paragraph 4 of the Limitation section.
Reporting biases	21	Present assessments of risk of bias due to missing results (arising from reporting biases) for each synthesis assessed.	Outlined in paragraph 6 of the Limitation section
Certainty of evidence	22	Present assessments of certainty (or confidence) in the body of evidence for each outcome assessed.	Outlined in paragraph 5 of the Limitation section
**DISCUSSION**			
Discussion	23a	Provide a general interpretation of the results in the context of other evidence.	Outlined in the Discussion section
	23b	Discuss any limitations of the evidence included in the review.	Outlined in the Limitation section
	23c	Discuss any limitations of the review processes used.	Outlined in the Limitation section

	23d	Discuss implications of the results for practice, policy, and future research.	Outlined in the Conclusion
**OTHER INFORMATION**			
Registration and protocol	24a	Provide registration information for the review, including register name and registration number, or state that the review was not registered.	The review was not registered.
	24b	Indicate where the review protocol can be accessed, or state that a protocol was not prepared.	The protocol was not prepared.
	24c	Describe and explain any amendments to information provided at registration or in the protocol.	Not applicable.
Support	25	Describe sources of financial or non-financial support for the review, and the role of the funders or sponsors in the review.	The authors received no support in any forms during the review process, as declared in the Methodological Quality and Data Extraction section of Methodology.
Competing interests	26	Declare any competing interests of review authors.	The authors have no competing interests, as declared in the Methodological Quality and Data Extraction section of Methodology.
Availability of data, code and other materials	27	Report which of the following are publicly available and where they can be found: template data collection forms; data extracted from included studies; data used for all analyses; analytic code; any other materials used in the review.	The data extracted from included studies, used for all analyses and materials used in the review are all publicly available as they were previously published data. The template data collection forms can be provided upon request.

From: **Page MJ, McKenzie JE, Bossuyt PM, Boutron I, Hoffmann TC, Mulrow CD, et al. The PRISMA 2020 statement: an updated guideline for reporting systematic reviews. BMJ 2021;372:n71. doi: 10.1136/bmj.n71**

For more information, visit: http://www.prisma-statement.org/

PRISMA 2020 Abstract Checklist

Section and Topic	Item #	Checklist item	Reported (Yes/No)
**TITLE**			
Title	1	Identify the report as a systematic review.	Yes
**BACKGROUND**			
Objectives	2	Provide an explicit statement of the main objective(s) or question(s) the review addresses.	Yes

**METHODS**			
Eligibility criteria	3	Specify the inclusion and exclusion criteria for the review.	No
Information sources	4	Specify the information sources (e.g. databases, registers) used to identify studies and the date when each was last searched.	No
Risk of bias	5	Specify the methods used to assess risk of bias in the included studies.	No
Synthesis of results	6	Specify the methods used to present and synthesise results.	Yes
**RESULTS**			
Included studies	7	Give the total number of included studies and participants and summarise relevant characteristics of studies.	Yes
Synthesis of results	8	Present results for main outcomes, preferably indicating the number of included studies and participants for each. If meta-analysis was done, report the summary estimate and confidence/credible interval. If comparing groups, indicate the direction of the effect (i.e. which group is favoured).	Yes
**DISCUSSION**			
Limitations of evidence	9	Provide a brief summary of the limitations of the evidence included in the review (e.g. study risk of bias, inconsistency and imprecision).	No
Interpretation	10	Provide a general interpretation of the results and important implications.	Yes
**OTHER**			
Funding	11	Specify the primary source of funding for the review.	No
Registration	12	Provide the register name and registration number.	No

From: **Page MJ, McKenzie JE, Bossuyt PM, Boutron I, Hoffmann TC, Mulrow CD, et al. The PRISMA 2020 statement: an updated guideline for reporting systematic reviews. BMJ 2021;372:n71. doi: 10.1136/bmj.n71**

For more information, visit: http://www.prisma-statement.org/

File S1: Search strategy

Search Hedge

(“drug coated balloon” OR “drug-coated balloon”) [all fields] OR (“drug eluting balloon” OR “drug-eluting balloon” OR “DEB”) [all fields] AND (“coronary” OR “cardiac”) [all fields] AND (“bifurcation lesion”) [all fields]

Limited to Human Studies, English Language
Search Performed: 1st December 2024

Databases

Ovid Medline (1946-Week 1 December 2024), Embase (1980-Week 1 December 2024), Cochrane Register of Controlled Trials (CCTR; December 2024), and Cochrane Database of Systematic Reviews (CDSR 2005-December 2024)

Figure S1: ROBINS-I Risk of Bias Assessment Table

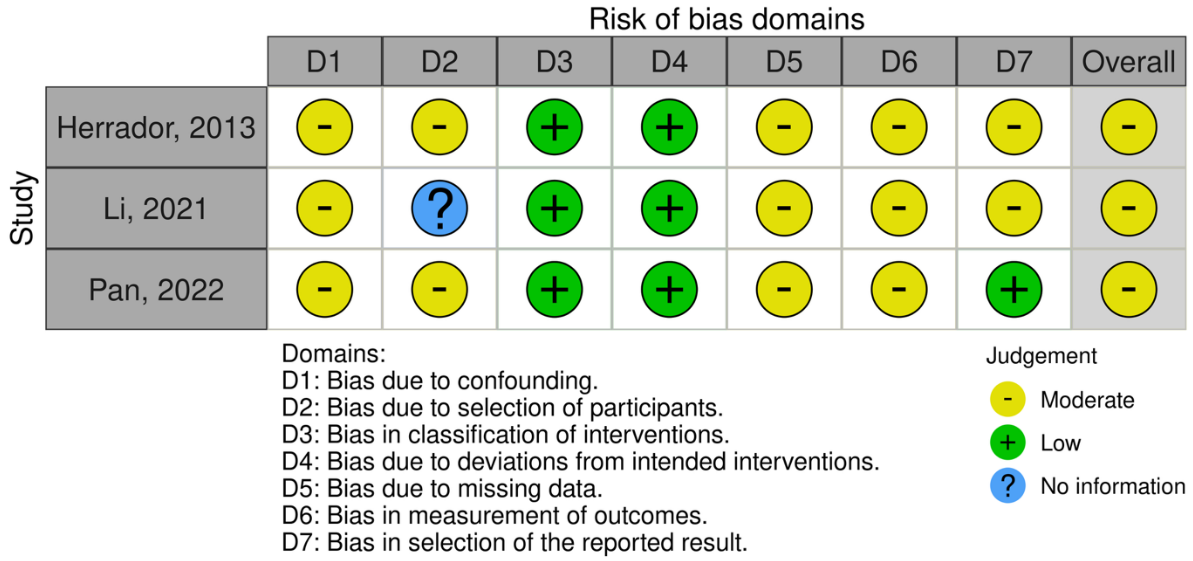

Figure S1: Risk of bias assessment of randomised control studies using the ROBINS-I tool

Figure S2: ROB-2 Assessment of Bias Table

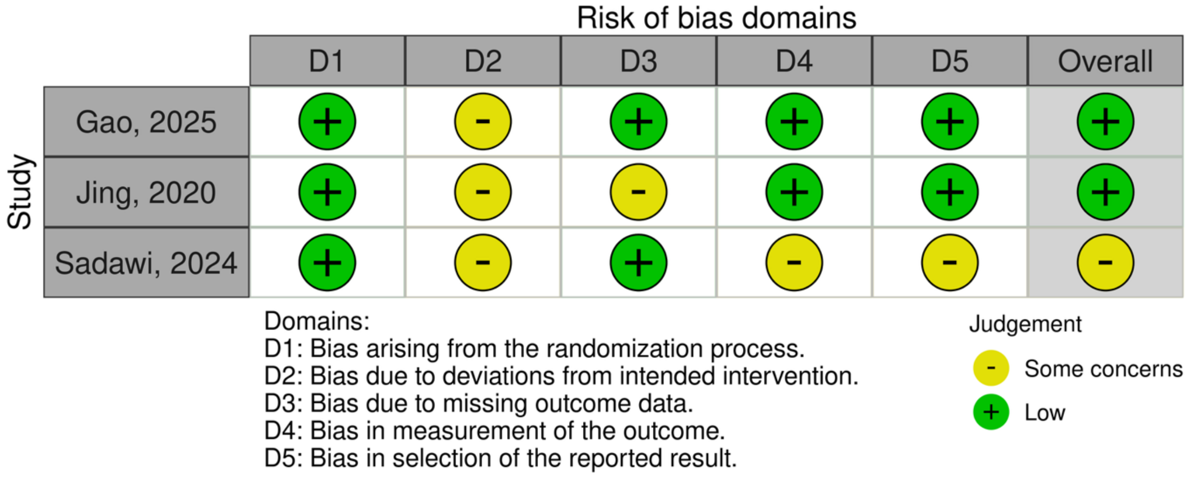

Figure S2: RIsk of bias assessment of non-randomised studies using the ROB-2 assessment tool

Figure S3: Assessment of Bias

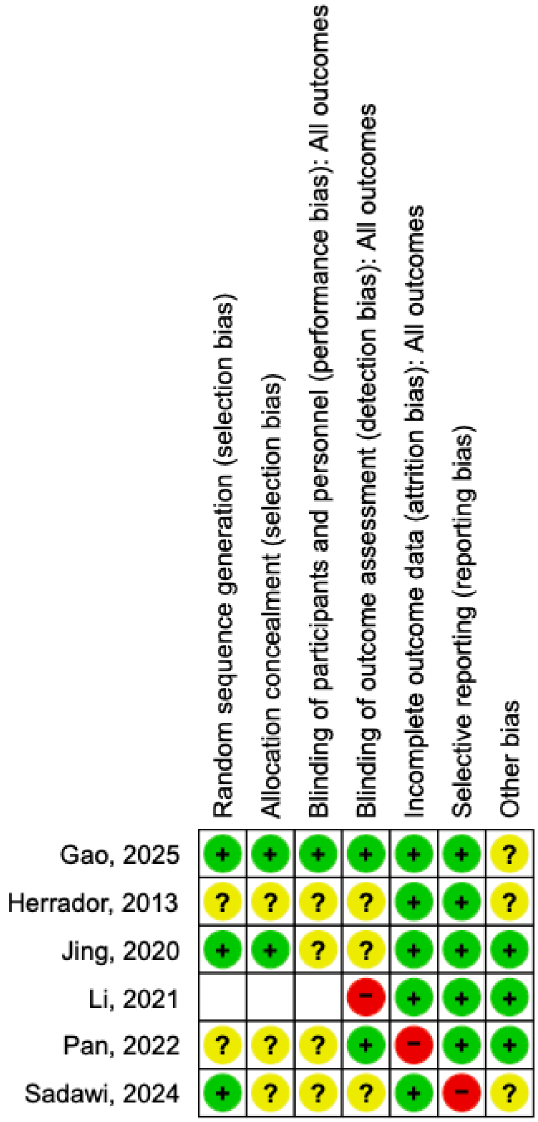

Figure S3: Risk of bias assessment for each study included in systematic review using the Cochrane Risk of Bias tool

Table S1: Patient Characteristics

Patient Characteristics	Age	Male	Diabetes	Smoking	HTN	Dyslipidaemia	Previous MI	Previous PCI	Stable angina pectoris	UA/NSTEMI	STEMI	LVEF (%)
**Herrador, 2013**	C: 61.9 ±10.8<br>E: 63.1 ± 11	C: 40/50 (80%)<br>E: 43/50 (86%)	C:16/50 (32%)<br>E:17/50 (34%)	C:25/50 (50%)<br>E:27/50 (54%)	C:31/50 (62%)<br>E:31/50 (62%)	C: 26/50 (52%)<br>E:30/50 (60%)	C:5/50 (10%)<br>E:7/50 (14%)	C:4/50 (8%)<br>E:5/50 (10%)	C:14/50 (28%)<br>E:10/50 (20%)	C:28/50 (56%)<br>E:30/50 (60%)	C:8/50 (16%)<br>E:10/50 (20%)	C:65.3±11.5<br>E:65.1±12
**Jing, 2020**	C: 61.8 ± 9.4<br>E: 59.9 ± 10.1	C: 71 (65.1)<br>E: 90 (79.7);	C: 38 (34.9)<br>E: 34 (30.1);	C: 56 (51.4)<br>E: 63 (55.8);	C: 65 (59.6)<br>E: 69 (61.1)	C: 28 (25.7)<br>E: 24 (21.2)	—	—	C: 4 (3.7)<br>E: 5 (4.4);	C: 100 (91.7)<br>E: 104 (92.0);	—	—
**Li, 2021**	C: 63±10<br>E: 64±11	C: 95/117 (81.2%)<br>E: 82/102 (80.4%	C: 32/117 (27.4%)<br>E: 33/102 (32.4%)	C: 63/117 (53.9%)<br>E: 58/102 (56.9%)	C: 65/117 (55.6%)<br>E: 52/102 (51%)	C: 28/117 (23.9%)<br>E: 24/102 (23.5%)	—	—	—	—	—	—
**Pan, 2022**	C: 64.48 ± 7.75<br>E: 63.75 ± 8.13,	C: 299 (75.13%)<br>E: 145 (72.86%),	C: 165 (41.46%)<br>E: 77 (38.69%),	C:138 (34.67%)<br>E: 72 (36.18%),	C: 205 (51.51%)<br>E: 107 (53.77%),	C: 119 (29.90%)<br>E: 54 (27.14%),	C: 26 (6.53%)<br>E: 11 (5.53%),	C: 74 (18.59%)<br>E: 31 (15.58%),	C: 162 (40.70%)<br>E: 75 (37.69%),	C: 236 (59.30%)<br>E: 124 (62.31%),	—	C: 59.38 ± 5.60<br>E: 59.64 ± 5.21
**Gao, 2025**	C: 63.6 ±10.5<br>E:63.8 ±10.6	C: 297 (75.6)<br>E: 305 (78.0)	C: 140 (35.6)<br>E: 147 (37.6)	C: 153 (38.9)<br>E: 164 (41.9)	C: 246 (62.6)<br>E: 257 (65.7)	C: 236 (60.1)<br>E: 251 (64.2)	C:39 (9.9)<br>E: 44 (11.3)	C: 102 (26.0)<br>E: 106 (27.1)	C: 24 (6.1)<br>E: 29 (7.4)	C: 334 (85)<br>E: 335 (85.7)	C: 23 (5.9)<br>E: 22 (5.6)	—
**Sadawi, 2024**	C: 60.5 ±7.2<br>E: 61.1±7.6	C: 23/30 (76.7%)<br>E: 22/30 (73.3%)	C: 13/30 (43.3%)<br>E: 12/30 (40.0%)	C: 14/30 (46.7%)<br>E: 12/30 (40.0%)	C: 18/30 (60.0%)<br>E: 19/30 (63.3%)	C: 22/30(73.3%)<br>E: 24/30 (80.0%)	C: 9/30 (30.0%)<br>E: 8/30 (26.7%)	.—	.—	C: 11 (36.6%)<br>E: 11 (36.6%)	C: 3 (10%)<br>E: 1 (3.3%)	.—

HTN: Hypertension; MI: Myocardial Infarction; PCI: Percutaneous Coronary Intervention; UA: Unstable angina; NSTEMI: Non-ST elevation myocardial infarction; STEMI: ST elevation Myocardial Infarctoin; LVEF: Left Ventricular Ejection Fraction; C: Contol; E: Experimental

Table S2: Summary of Study outcomes

Study characteristics	Follow up	TLR	Non fatal MI	Cardiac Death	LLL MB at follow up (mm)	LLL SB at follow up (mm)
**Herrador, 2013**	12 months	C: 11/50, E: 6/50	C: 1/50, E:0/50	C: 0/50, E: 0/50	C: 0.62 ± 0.7, E: 0.49±0.6	C: 0.4±0.5, E: 0.09 ± 0.4
**Jing, 2020**	9 months	C: 0/109, E: 0/113	C: 1/109, E: 0/113	C: 0/109, E: 0/113	E: 0.12 (–0.09–0.35)<br>C: 0.08 (–0.10–0.38)	E: -0.06 ± 0.32<br>C: 0.18 ± 0.34
**Li, 2021**	12 months	—	C: 7/117, E 3/102	C: 1/117, E 1/102	C: 0.13±0.14<br>E: 0.12±0.14	C: 0.19±0.25<br>E: 0.11±0.18
**Pan, 2022**	12 months	At 12mo<br>C: 27/398, E: 3/199	At 12mo<br>C: 8/398, E: 2/199	At 12mo<br>C: 9/398, E: 2/199	At 2 years<br>C: 0.22 ± 0.42, E: 0.25 ± 0.48	At 2 years<br>C: 0.42 ± 0.62, E: 0.13 ± 0.42
**Gao, 2025**	12 months	C: 6/393, E: 5/391	C: 14/393, E: 4/391	C: 2/393, E: 4/391	—	—
**Sadawi, 2024**	6 months	C: 2/30, E:0/30	C: 2/30, E: 1/30	—	—	—
TLR: Target Lesion Revascularisation; MI: Myocardial Infarction; LLL: Late Lumen Loss; MB: Main Branch; SB: Side Branch						

Figure S4: Funnel Plot

Figure S4A: Cardiac Mortality at Follow Up

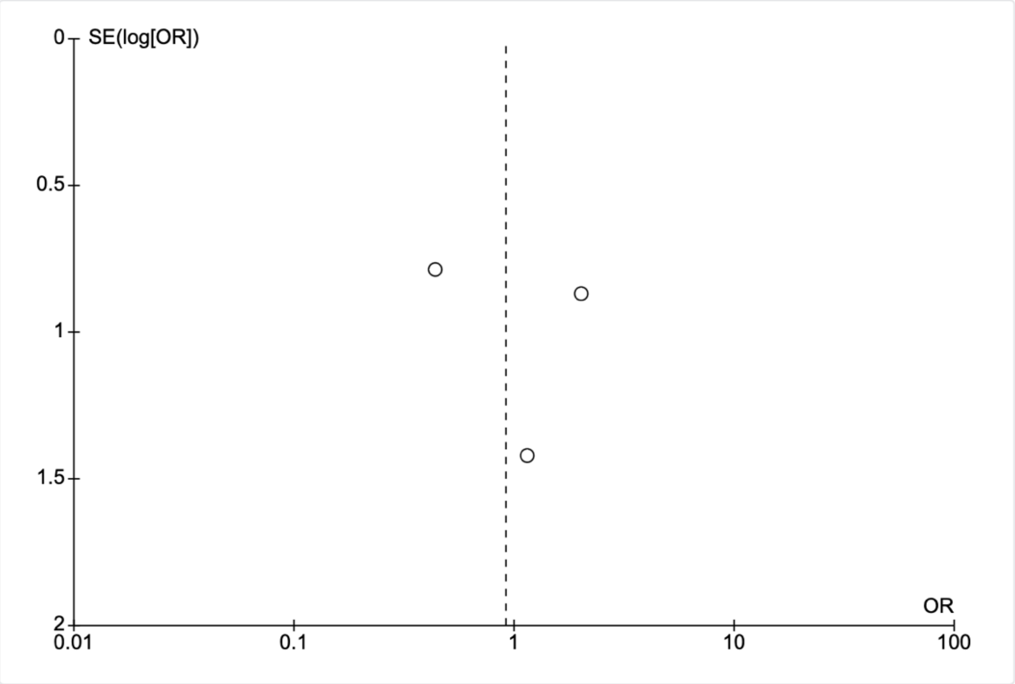

Figure S4A: Funnel plot assessing publication bias for studies reporting 12-month cardiac mortality odds ratios (OR) following drug-coated balloon (DCB) use in side-branch treatment. The symmetrical distribution indicates low risk of publication bias at 12-month follow-up.

Figure S4B: Late Lumen Loss MB at Follow Up

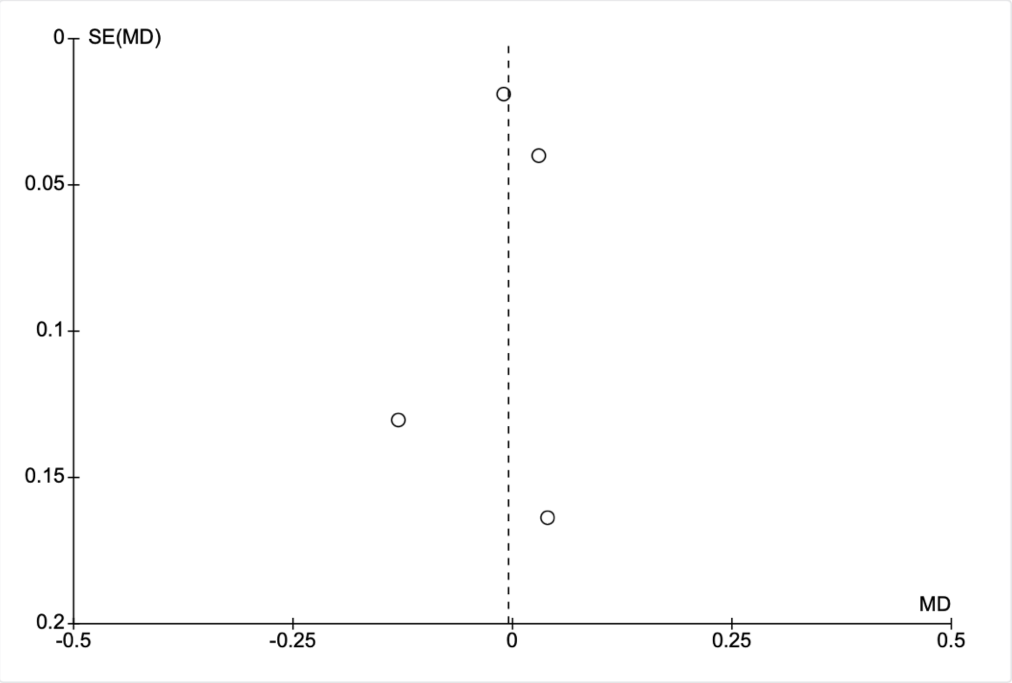

Figure S4B: Funnel plot assessing publication bias for studies reporting 12-month late lumen loss (LLL) in the main branch (MB) odds ratios following drug-coated balloon (DCB) use in side-branch treatment. The symmetrical distribution indicates low risk of publication bias at 12-month follow-up.

Figure S4C: Late Lumen Loss SB at Follow Up

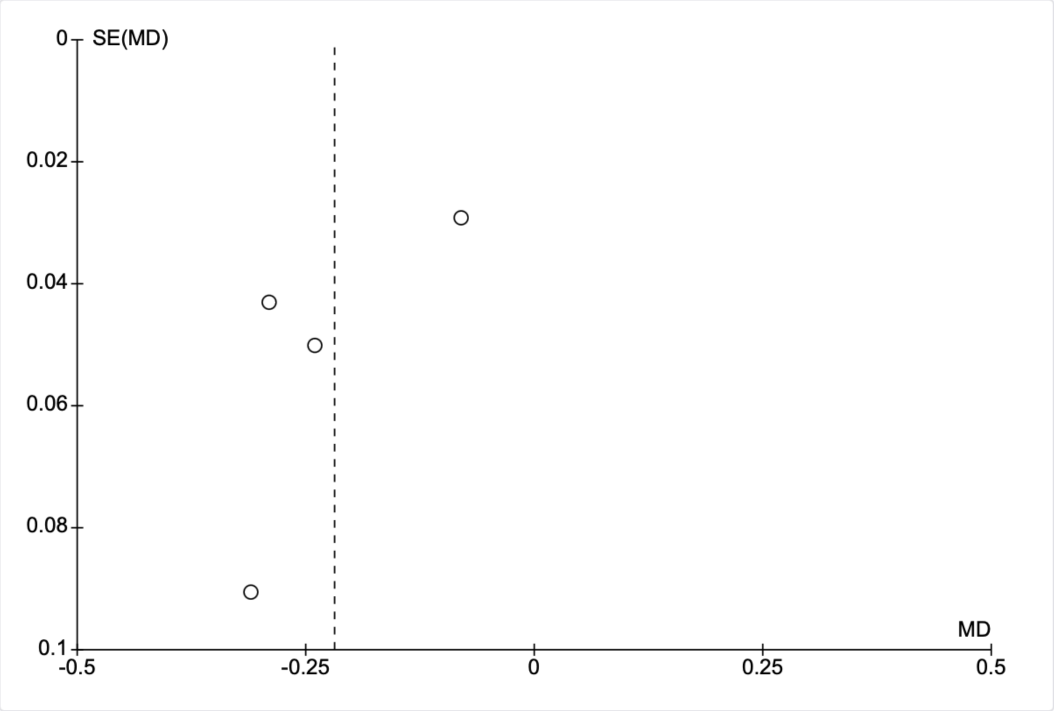

Figure S4C: Funnel plot assessing publication bias for studies reporting 12-month late lumen loss (LLL) in the side branch (SB) odds ratios (OR) following drug-coated balloon (DCB) use in side-branch treatment. The asymmetrical distribution indicates moderate risk of publication bias at 12-month follow-up.

Figure S4D: Myocardial Infarction at Follow Up

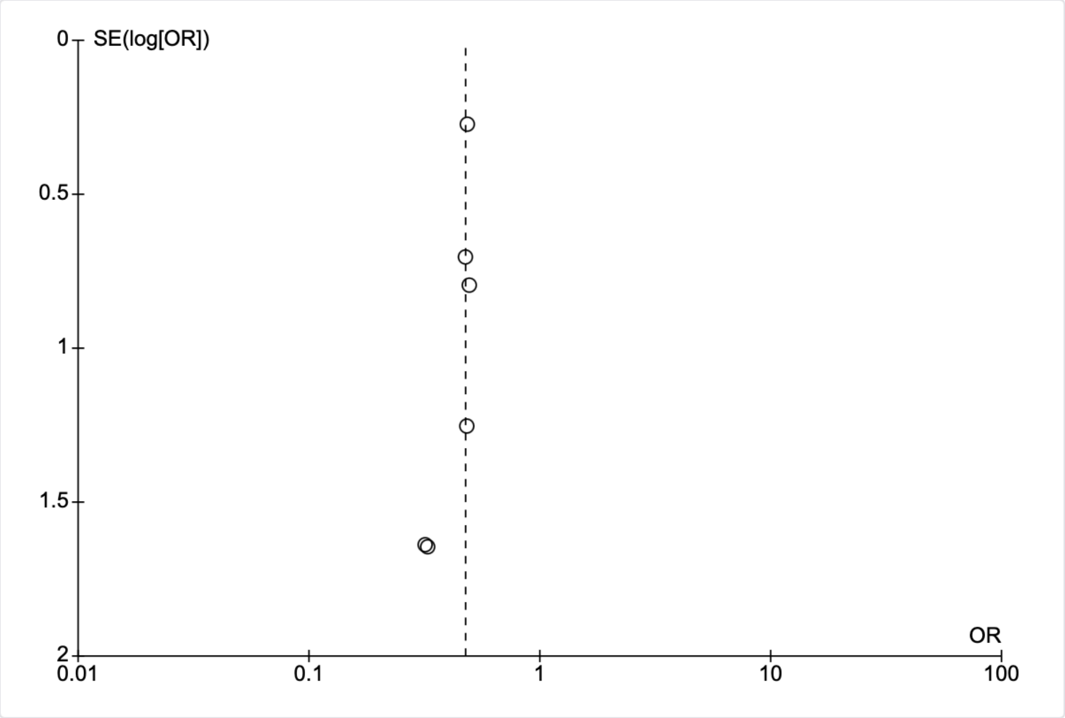

Figure S4D: Funnel plot assessing publication bias for arm studies reporting 12-month myocardial infarction odds ratios (OR) following drug-coated balloon (DCB) use in side-branch treatment. The symmetrical distribution indicates low risk of publication bias at 12-month follow-up.

Figure S4E: Target Lesion Revascularisation at Follow Up

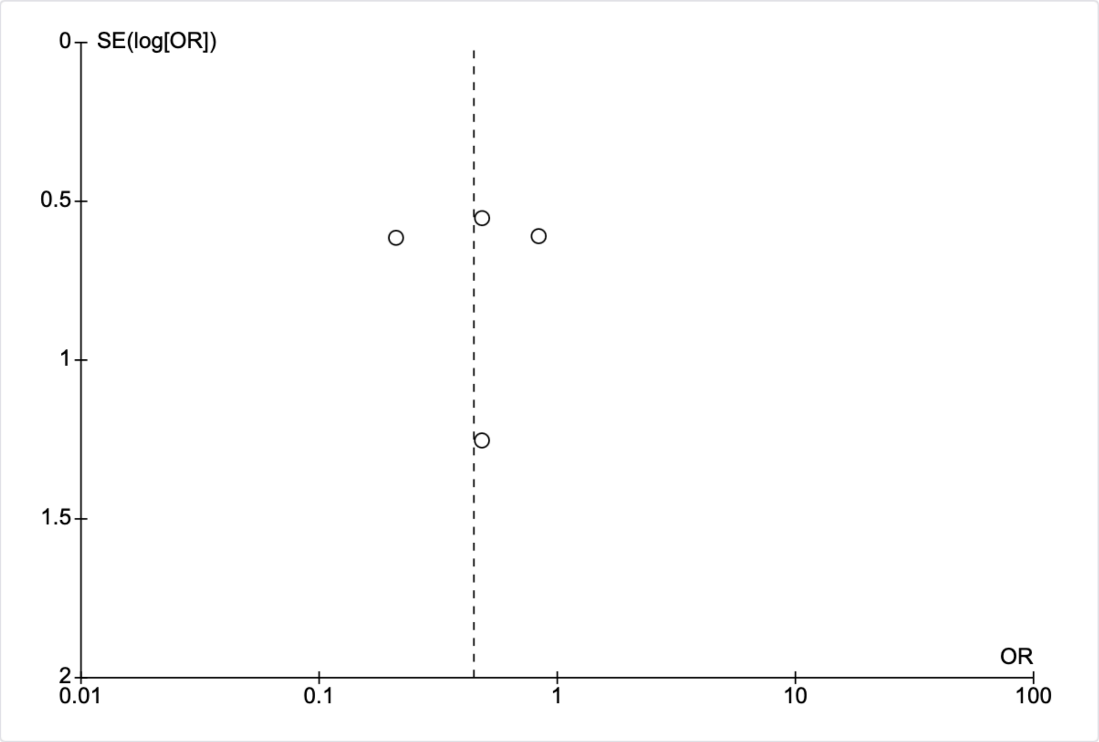

Figure S4E: Funnel plot assessing publication bias for arm studies reporting 12-month target lesion revascularisation odds ratios (OR) following drug-coated balloon (DCB) use in side-branch treatment. The symmetrical distribution indicates low risk of publication bias at 12-month follow-up.

Figure S5. Proportional Meta-analysis

Figure S5A: Pooled MI Rate at 12 Months.

Study	Events	Total	Weight	IV, Random, 95% CI
Worthley, 2015	1	35	2.6%	0.03 [0.00; 0.15]
Pellegrini, 2023	0	50	3.6%	0.00 [0.00; 0.07]
Lazar, 2024	5	194	14.0%	0.03 [0.01; 0.06]
Teo 2024	3	363	26.2%	0.01 [0.00; 0.02]
Herrador, 2013	0	50	3.6%	0.00 [0.00; 0.07]
Li, 2021	3	102	7.4%	0.03 [0.01; 0.08]
Pan, 2022	2	199	14.4%	0.01 [0.00; 0.04]
Gao, 2025	4	391	28.2%	0.01 [0.00; 0.03]
**Total (95% CI)**		**1384**	**100.0%**	**0.01 [0.00; 0.02]**
**Prediction interval**				**[0.00; 0.02]**

Heterogeneity: $Tau^2 = 0$; $Chi^2 = 6.56$, df = 7 (P = 0.4766); $I^2 = 0.0\%$

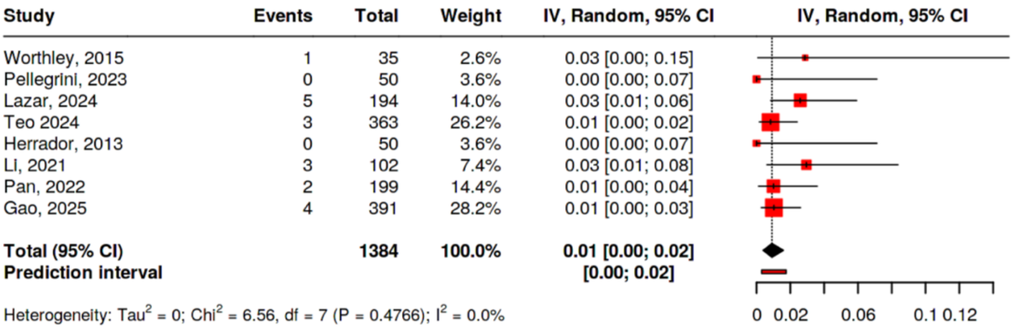

Figure S5A: Single-arm proportional meta-analysis of 12-month Myocardial infarction (MI) rates after drug-coated balloon (DCB) angioplasty in side-branch (SB) treatment. The pooled MI incidence is 1% (95% CI: 0-2%; prediction interval 0-2%), with negligible heterogeneity ($I^2 = 0\%$, $Tau^2 = 0$).

Figure S5B: Pooled TLR Rate at 12 Months.

Study	Events	Total	Weight	IV, Random, 95% CI
Worthley, 2015	1	35	6.7%	0.03 [0.00; 0.15]
Pellegrini, 2023	1	50	8.6%	0.02 [0.00; 0.11]
Lazar, 2024	8	194	17.2%	0.04 [0.02; 0.08]
Teo 2024	5	363	20.6%	0.01 [0.00; 0.03]
Herrador, 2013	6	50	8.6%	0.12 [0.05; 0.24]
Pan, 2022	3	199	17.4%	0.02 [0.00; 0.04]
Gao, 2025	5	391	20.9%	0.01 [0.00; 0.03]
**Total (95% CI)**		**1282**	**100.0%**	**0.02 [0.01; 0.04]**
**Prediction interval**				**[0.00; 0.08]**

Heterogeneity: $Tau^2 = 0.0024$; $Chi^2 = 15.46$, df = 6 (P = 0.0169); $I^2 = 61.2\%$

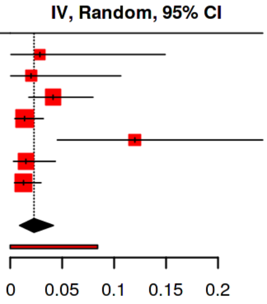

Figure S5B: Single-arm proportional meta-analysis of 12-month target lesion revascularisation (TLR) rates after drug-coated balloon (DCB) angioplasty in side-branch (SB) treatment. The pooled TLR incidence is 2% (95% CI: 1-4%; prediction interval 0-8%), with moderate heterogeneity ($I^2 = 61.2\%$, $Tau^2 = 0.0024$).

Figure S5C: Pooled Cardiac Mortality at 12 Months.

Study	Events	Total	Weight	IV, Random, 95% CI
Worthley, 2015	1	35	2.6%	0.03 [0.00; 0.15]
Pellegrini, 2023	0	50	3.8%	0.00 [0.00; 0.07]
Lazar, 2024	6	194	14.2%	0.03 [0.01; 0.07]
Teo 2024	2	363	25.9%	0.01 [0.00; 0.02]
Herrador, 2013	0	50	3.8%	0.00 [0.00; 0.07]
Li, 2021	1	102	7.6%	0.01 [0.00; 0.05]
Pan, 2022	2	199	14.5%	0.01 [0.00; 0.04]
Gao, 2025	4	391	27.7%	0.01 [0.00; 0.03]
**Total (95% CI)**		**1384**	**100.0%**	**0.01 [0.00; 0.01]**
**Prediction interval**				**[0.00; 0.02]**

Heterogeneity: $Tau^2 < 0.0001$; $Chi^2 = 7.16$, df = 7 (P = 0.4121); $I^2 = 2.3\%$

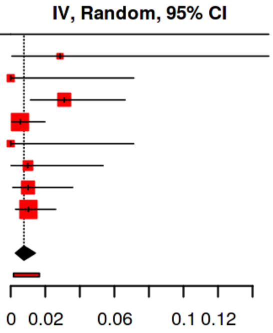

Figure S5C: Single-arm proportional meta-analysis of 12-month cardiac mortality rates after drug-coated balloon (DCB) angioplasty in side-branch (SB) treatment. The pooled cardiac mortality incidence is 1% (95% CI: 0-1%; prediction interval 0-2%), with minimal heterogeneity ($I^2 = 2.3\%$, $Tau^2 < 0.0001$).

Table S3: Single Arm Study Characteristics

Table S3: Table of study characteristics for single arm studies included in meta-analysis

Study	Intervention	Inclusion/Exclusion Criteria	Stent	Drug coated balloon	Primary End-point	Secondary Endpoint	Follow up
**Berland, 2015** prospective, non-randomised, multicentre, interventional study, 8 centres May 2012 - July 2013	Single arm study, DES in MB, DEB in SB (n:52)	(1) M+F >18 years, (2) clinical evidence of ischaemic heart disease, stable or unstable angina with signs of ischaemia, silent ischaemia, or a positive functional test, (3) Lesion length ≤6 mm in SB (4) reference vessel diameter between 2.5 - 3.5 mm for MB and 2.0 - 3.0 mm for SB.	'Nile PAX', paclitaxel DES	DANUBIO paclitaxel-eluting PTCA balloon	6 month LLL at SB ostium	MB LLL, binary restenosis of the SB & MB, CD-TLR for MB + SB, MACE in MB and SB (MI, TLR, cardiac death), angiographic success	Angio follow up at 6mo. Clinical follow up at 1, 3, 6 & 12mo. 4/52 (4%) lost
**Worthley, 2015** prospective, multi-center, single arm pilot study, 5 centres January 2011 and August 2012	Single arm study, DES in MB, DEB in SB (n:35)	(1) Single target lesion or two lesions (target and non-target) located in separate coronary arteries, (2) target lesion is single de novo CBL with a reference vessel diameter of 2.0 to 4.0 mm in the MB and SB with a main branch stenosis of ≥50% and 100%, (3) target vessel thrombolysis in myocardial infarction (TIMI) flow ≥2 was required.	Everolimus-eluting stent	Pantera Lux paclitaxel coated	9 month SB LLL	1. Device & procedural success (death, MI or TLR during the hospital stay)<br>2. lesion success (not needing other lesion treatment during the index procedure).	Clinical assessments at 1, 6, 9 & 12mo. Angiogram + IVUS at 9mo. 0 lost to f/up
**Kasboui, 2023** prospective non-randomized	Single arm study, DES in MB, DEB in SB (n:45)	(1) indication of a PCI of a de novo true bifurcation lesion Medina (1.1.1) or Medina (0.1.1). Angiographic exclusion criteria were: SB diameter <	(Synergy XDTM®, Synergy MegatronTM	The DCB Agent (Boston	Composite of TLF at 6mo including cardiac death,	1. technical success (PCI without an additional DES at the level of the SB ostium,	6 month. 3 (6.7%) lost (non-cardiac death)

interventional single- center study September 2020 to March 2022,		2 mm or > 3.5 mm, ostial SB stenosis ≤50 %, ostial SB lesion length > 20 mm, ostial SB severe calcifications, target lesion location in a vessel which has a bypass, in-stent restenosis or stent thrombosis.	® and Resolute onyxTM®	Scientific)- Paclitaxel	TVMI or CD-TLR	2. clinical success (technical success associated with the absence of severe complications during in-hospital phase).	
**Pellegrini, 2023** Substudy of the HYPER trial, a prospective, single-arm, multicentre, pilot study April 2019 to December 2020	Single arm study, DES in MB, DEB in SB (n:50)	Patients with chronic or acute coronary syndromes and a CBL involving the SB and at least one of the main vessel (MV) or the MB, i.e. 1.0.1, 0.1.1 or 1.1.1 lesions according to the Medina classification	DES stent	Restore DCB (Cardionovum GmbH, Germany)- Paclitaxel	Cardiac death, TVMI and CD-TLR	1. Procedural success 2. Peri-procedural MI, 3. Individual MACE (cardiac death, any TV-MI excluding peri-procedural MI, ID-TLR) at 1-year follow-up. 4. Any definite/probable DES- or DCB-treated segment thrombosis or occlusion at 1-year follow-up.	12 months. 0 lost to f/up
**Lazar, 2024** prospective, investigator-driven, multicenter clinical registry. Substudy of eaST-BoUrne registry, 38 centres ND- Registry published July 2023	A)DES in MB, DEB in SB (n:50) B) Full DES approach	Substudy of EASTBOURNE registry of patients with Medina lesions 1.1.1/1.1.0/1.0.1/0.1.1/0.1.0. Target lesion/vessel with any 1 of the following characteristics were excluded: (a) unsuccessful predilatation of the target lesion, with persisting residual stenosis >50%; (b) severe calcification of the target vessel, either at the lesion site or proximal to the lesion; (c) highly tortuous culprit vessels; and (d) visible thrombus at the lesion site, not treatable with manual aspiration.	DES stent	Sirolimus-DCB (Magic Touch, Concept Medical, India)	TLR at 12 months	1. Angiographic success (residual stenosis of <50% without periprocedural complications) 2.procedural success (angiographic success in the absence of in-hospital MACE including death, MI, TLR, stroke, vascular access site complications, and contrast nephropathy	12 month. 7% lost to follow up.

**Teo, 2024** retrospective, single-centre, observational study 1 July 2021 to 30 June 2022	Single arm study, DES in MB, DEB in SB (n:401)	Patients >18, admitted to National Heart Institute Kuala Lumpur with chronic or acute coronary syndrome. Those treated with DES and DCB were enrolled following PCI treatment.	Everolimus (DES) in 202 (50.4%), Zotarolimus (DES) in 116 (28.9%)	Paclitaxel-eluting balloons 99% of DCBs	TLF in the DES/DCB-treated segment. TLF (cardiac death, TVMI and ID-TLR)	All-cause mortality, major adverse cardiovascular events (MACE) and TLF predischarge or within 30 days of the procedure.	12 month. 38/401 (9.5%) lost

DES: Drug eluting stent; MB: Main Branch; MV: Main Vessel; POBA: Plain Old Balloon Angioplasty; SB: Side Branch; DEB: Drug-eluting balloon; LLL: Late Lumen Loss; MACE: Major Adverse Cardiac Event; US: Ultrasound, IVUS: Intravascular ultrasound; PCI: Percutaneous Coronary Intervention; MI: Myocardial infarction; CBL: Coronary bifurcation lesion; TLR: Target Lesion Revascularisation; TVR: Target Vessel Revascularisation; PTCA: Percutaneous transluminal coronary angioplasty; TLF: Target Lesion Failure; TVMI: Target Vessel Myocardial Infarction; CD-TLR: Clinically Driver Target Lesion Revascularisation; M: Male; F: Female; mo: months; TVR: Target Vessel Revascularisation.
